# Non-peptide guided auto-secretion of recombinant proteins by super-folder green fluorescent protein in *Escherichia coli*

**DOI:** 10.1038/s41598-017-07421-3

**Published:** 2017-08-01

**Authors:** Zhen Zhang, Rongxing Tang, Dewu Zhu, Wenfeng Wang, Li Yi, Lixin Ma

**Affiliations:** Hubei Collaborative Innovation Center for Green Transformation of Bio-resources, Hubei Key Laboratory of Industrial Biotechnology, College of Life Sciences, Hubei University, Wuhan, People’s Republic of China

## Abstract

Protein secretion in *Escherichia coli* is usually led by a signal peptide that targets the protein to specific secretory pathways. In this study, we demonstrated that the superfolder green fluorescent protein (sfGFP) could be served as a non-signal peptide to guide protein auto-secretion in *E*. *coli*. This auto-secretion was characterized as a three-step process through the sub-cellular localization analysis: inner membrane trans-location followed by anchoring at outer membrane, and then being released into culture media. We further determined that the beta-barrel structure and net negative charges of sfGFP played important roles in its auto-extracellular secretion property. Using sfGFP as a carrier, heterologous proteins ranging from peptide to complex protein, including antibacterial peptide PG4, endo-beta-N-acethylglucosamindase H (Endo H), human arginase-1 (ARG1), and glutamate decarboxylase (GAD) were all successfully expressed and secreted extracellularly when fused to the carboxyl end of sfGFP. Besides facilitating the extracellular secretion, sfGFP fusion proteins can also be correctly folded and formed the active complex protein structure, including the trimetric human ARG1 and homo-hexametric GAD. This is the first report that sfGFP can guide the secretion of recombinant proteins out of the cells from cytoplasm in *E*. *coli* without affecting their conformation and function.

## Introduction

As one of the best-characterized host cells, *Escherichia coli* has been widely used as a cell factory for the production of heterologous proteins^1, 2^. Heterologous protein expression in *E*. *coli* can be categorized as cytoplasmic and extracellular production. The secretory production of heterologous proteins avoids the cell lysis step, therefore provides simpler protein purification process and lower production expense than the intracellular production, which is preferred in the industrial scale production^3–5^. Several protein secretory systems from the cytosol into the periplasm or the extracellular milieu have been identified in *E*. *coli* ^6^, and Type I, Type II, and Type V secretion systems are the most commonly used ones.

In the Type I secretion system (T1SS), an ATP binding cassette (ABC) transporter recognizes the C-terminal signal peptide of a target protein, facilitating its recognition by *E*. *coli* α-haemolysin (HlyA) followed by transportation directly from the cytoplasm to the culture medium^7–9^. Compared to the T1SS, type II secretion system (T2SS) can be separated as two steps, periplasmic trans-location and extracellular transport. Periplasmic trans-location is mediated by Sec-dependent trans-location pathway (Sec pathway) or Twin-arginine trans-location pathway (Tat pathway). Sec pathway is guided by the Sec signal peptide and used for the trans-location of unfolded proteins, while Tat pathway is guided by the Tat signal peptide and used for the secretion of folded proteins^10, 11^. After the protein traverses the inner membrane, the signal peptide that is originally located at the N-terminus of the protein is removed by a signal peptidase^12^. Followed by the periplasmic trans-location, the extracellular release of the protein is carried out by methods that disturbed the permeability of outer membrane, such as osmotic pressure or chemical molecule^12^. Type Va is a well characterized model within Type V pathways which shares a distinctive organization of the fusion complex: a Sec signal peptide at N-terminus, the target protein at middle, and a beta-barrel structure at C-terminus^13, 14^. The Sec signal peptide guides the trans-location of the whole protein complex to the inner membrane in an unfolded status. Then the beta-barrel (C terminal recognition sequence: X-Z-X-Z-X-Z-Y-Z-F/W; X: hydrophobic amino acid, Z: any amino acids)^15, 16^ is recognized and folded on the outer membrane, facilitating the secretion of target protein out of the cells^17^.

Unconventional secretion pathway also exists in *E*. *coli*. For example, catalytic domain of a cellulase (Cel-CD) from *Bacillus* sp. can be secreted into the medium of the recombinant *E*. *coli* BL21(DE3) in large quantities without its native signal peptide during a two-step process. The N-terminal sequence of the full length Cel-CD and its three dimensional structure of protein play a crucial function in the secretion, and can serve as a carrier for the secretion of heterologous target proteins out of *E*. *coli* in an unraveled way^18^.

Wild-type GFP (wtGFP) is a ~26 kDa protein that emits green fluorescent light when exposed to the light ranging from blue to ultraviolet spectral spectrum^19, 20^. A variety of GFP mutants have been constructed to make it more soluble and stable with brighter emission than the wild-type version^21^, thus expanding its application as reporter proteins in developmental and cell biology studies^22, 23^. Recently, a mutant, called superfolder green fluorescent protein (sfGFP), was developed, which has a very stable beta-barrel structure and superior features among GFP mutants, such as high solubility, bright fluorescence, fast folding ability, and high resistance to denaturants^24^. Utilizing its property of high solubility, sfGFP has been developed as fusion proteins to enable proper folding of heterologous proteins in *E*. *coli* ^25^.

**Figure 1:**
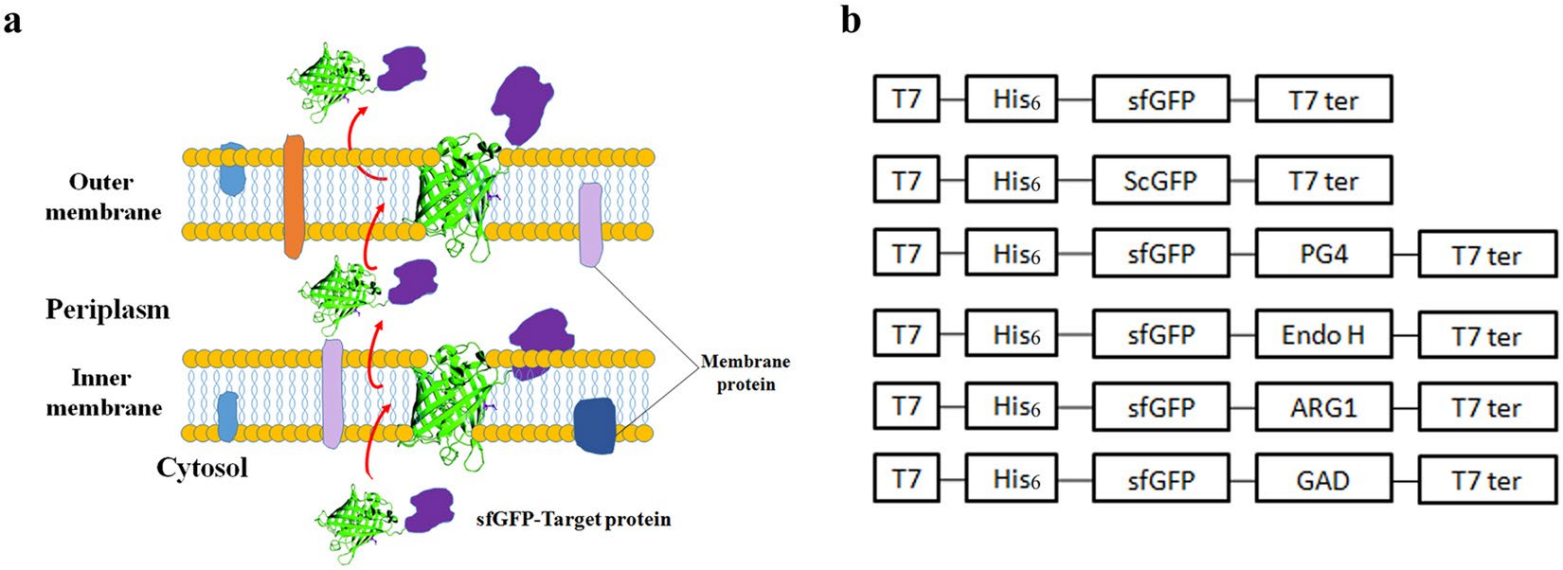
Diagrammatic sketch. (**a**) Possible secretory mechanism ofthe sfGFP fusion protein; (**b**) Constructs generated in this study; T7: T7 promoter; His_6_: 6 × His epitope tag; sfGFP: superfolder green fluorescent protein; scGFP: super charging green fluorescent protein; PG4: antibacterial peptide PG4; Endo H: endo-beta-N-acethylglucosamindase H; ARG1: human arginase-1; GAD: Glutamate decarboxylase; T7 ter: T7 terminator.

In this study, sfGFP was identified to enable auto-extracellular secretion in *E*. *coli* strains. Further analysis demonstrated that the sfGFP auto-secretion was divided into three steps: inner-membrane translocation, outer membrane translocation, and extracellular secretion (Fig. 1). In addition, our results suggested that the beta-barrel structure and net negative charges of sfGFP played important roles in the auto secretion process. Base on this interesting property of sfGFP, a new strategy, *E*. *coli* sf*G*FP mediated *P*rotein *S*ecretion *S*ystem (EGPSS), was developed in *E*. *coli*, which used sfGFP as N-terminal leading protein for the secretory expression of heterologous protein. The EGPSS was then used to express and secret various proteins, ranging from short polypeptides to protein complex, such as antibacterial peptide PG4, endo-beta-N-acethylglucosamindase H (Endo H), trimetric human arginase-1 (ARG1), and hexametric glutamate decarboxylase (GAD). Interestingly, fusion enzymes maintained enzymatic activities. The EGPSS was also tested in the fed-batch fermentation, confirming its high efficiency and stability for future industrial applications.

## Results

### Identification and characterization of the auto-secretion of sfGFP in *E*. *coli*

**Figure 2:**
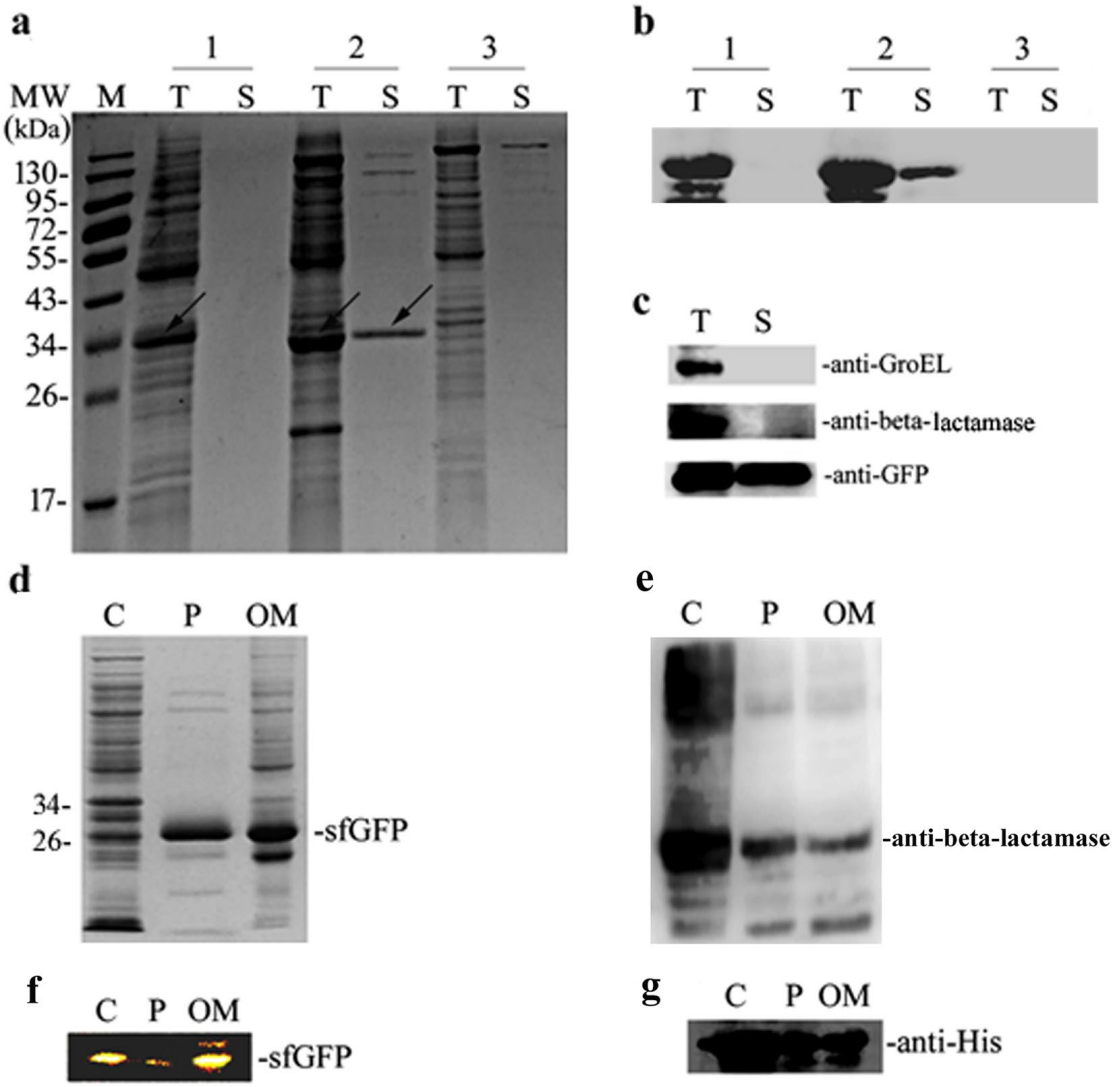
The characterization of sfGFP secretion in *E*. *coli*. (**a**) SDS-PAGE analysis the expression of sfGFP and scGFP in *E*. *coli*; 1: scGFP expressed in *E*. *coli* strain *Rosetta Blue*; 2: sfGFP expressed in *E*. *coli* strain *Rosetta Blue*; 3: negative control (plasmid pET23a) expressed in *E*. *coli* strain *Rosetta Blue*; (**b**) Western-Blot analysis of the expression of the sfGFP and scGFP in *E*. *coli*; the analysis was carried out using the anti-GFP monoclonal antibody; (**c**) Western-Blot analysis of the extracellualr secretion of sfGFP in *E*. *coli*; anti-GroEL:cytoplasmic protein GroEL monoclonal antibody; anti-beta lactamase: periplasmic protein beta lactamase monoclonal antibody; anti-GFP: GFP monoclonal antibody; (**d**) SDS-PAGE analysis of the sub-fraction of sfGFP in *E*. *coli*; (**e**) Western Blot analysis of the sub-fraction of sfGFP in *E*. *coli*; and the analysis was carried out using the periplasmic protein beta-lactamase monoclonal antibody; (**f**) Native-PAGE analysis of the sub-fractions of sfGFP in *E*. *coli*; (**g**) Western Blot analysis of sub-fractions of sfGFP in *E*. *coli*; anti-His: 6 × His monoclonal antibody. The lane markers were in Fig. 2, T: total protein; S: supernatant of cell culture media; C: cytoplasmic protein; P: periplasmic protein; OM: outer membrane protein.

The auto-secretion property of sfGFP was coincidentally discovered when it was over-expressed in the *E*. *coli* strain *Rosetta Blue* under IPTG induction with partial sfGFP (about 15%) detected in the culture medium (Fig. 2a). This secretion of sfGFP was further confirmed by immuno-blotting using anti-6 × His antibody against the 6 × His-tagged sfGFP (Fig. 2b).

To exclude the possibility that the secretion of sfGFP to the medium was caused by cell lysis, the secretion of sfGFP, GroEL (a well-defined cytoplasm protein), and beta -lactomase (a well-defined periplasmic enzyme) were evaluated in *Rosetta Blue* cells. Interestingly, sfGFP was detected in the culture medium, while endogenous protein, such as GroEL and beta -lactamase, were not detected in the culture medium (Fig. 2c). Moreover, the sfGFP secretion was detected not only in *E*. *coli* strain *Rosetta Blue* cells, but also in BL21(DE3) (data not shown) and DH10β cells, confirming the auto-secretion property of sfGFP in *E*. *coli*.

As a Gram-negative bacterium, *E*. *coli* contains inner membrane and outer membrane. Therefore it is interesting to determine the sub-cellular localization of sfGFP during its extracellular secretion process in *E*. *coli*. Interestingly, sfGFP was not only detected in culture media, but also in cytoplasm, periplasm, and outer membrane (Fig. 2d,e). Moreover, the green fluorescence in cytoplasm, periplasmic space, and outer membrane was also observed using fluorescence microscope, indicating that the sfGFP was well folded at these localizations (Fig. 2f). Based on these findings, we proposed that sfGFP maintained its folded structure during the extracellular secretion process, and this process could be divided into three processes: inner-membrane translocation, outer membrane translocation, and extracellular secretion.

Generally, protein transportation was led by the N-terminal signal peptides, such as Sec signal peptide or Tat signal peptide^11^. Interestingly, no potential signal peptide was identified in the sfGFP through analysis using the SignalP4.1 online software (http://www.cbs.dtu.dk/services/SignalP/) (Suppl. Figure 2a). Detection of His_6_-sfGFP from the culture medium using Western-blot analysis confirmed that the N-terminal sequence was kept intact in the secreted sfGFP (Fig. 2b). Moreover, the N-terminal intact sfGFP was also detected in cytoplasm, periplasmic space, and outer membrane by Western blotting using anti-6 × His antibody (Fig. 2g). sfGFP without 6 × His Tag could also auto-secrete expression in *E*. *coli* strain *Rosetta Blue* (Suppl. Figure 2b), suggesting that the whole secretion process of sfGFP might be N-terminal signal peptide unrelated.

### The effect of beta-barrel structure on sfGFP auto-secretion

**Figure 3:**
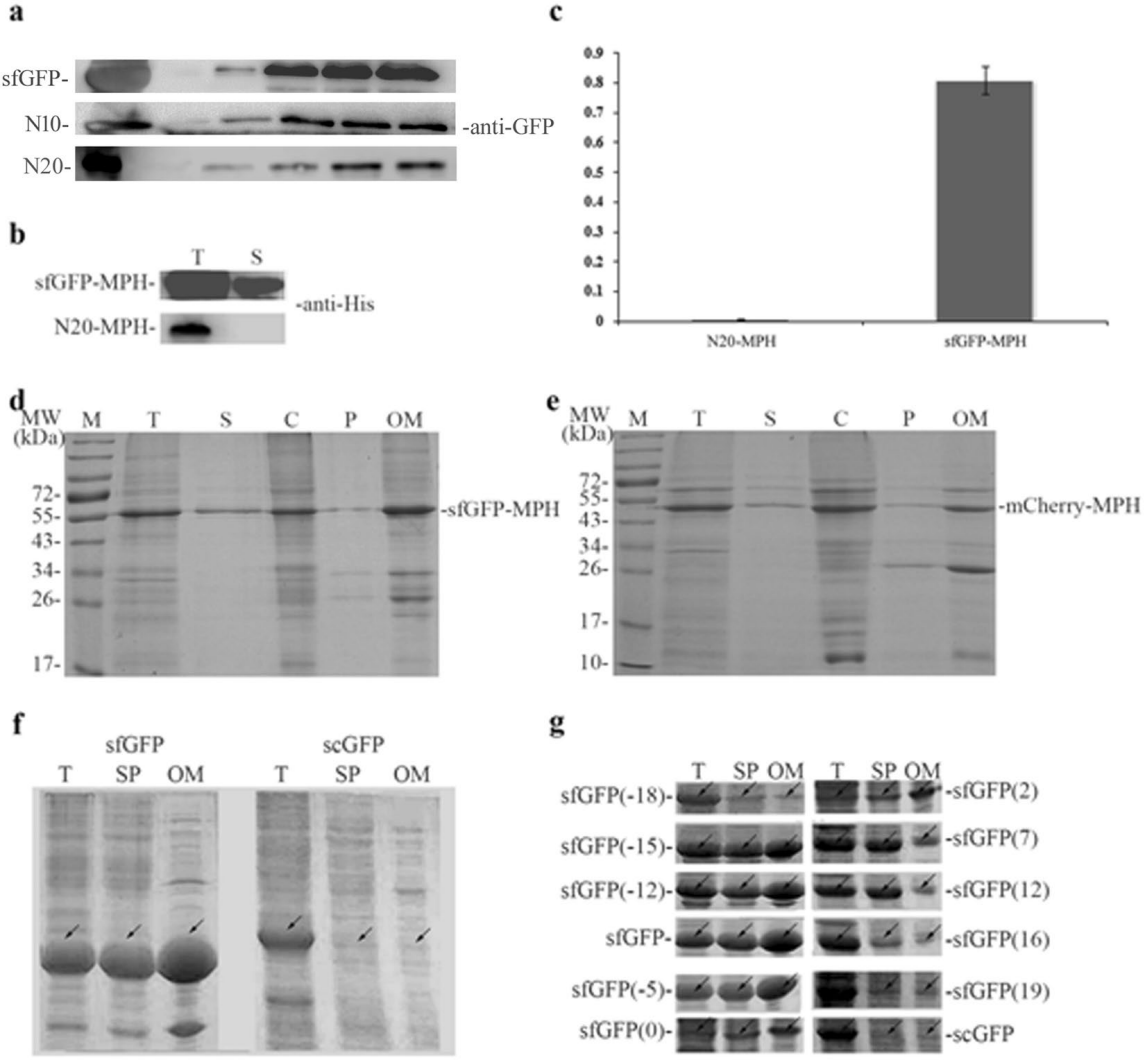
Analysis the auto secetion mechanism of sfGFP in *E*. *coli*. (**a**) Western blot annalysis of the extracellular expression of sfGFPs in *E*. *coli*; N10: mutant of sfGFP with deletion of 10 amino acids at its N-terminus; N20: mutant of sfGFP with deletion of 20 amino acids at its N-terminus; anti-GFP: GFP monoclonal antibody; all samples were tested by using GFP monoclonal antibody; (**b**) Western blot annalysis of the extracellualr secretion of sfGFP-MPH and N20-MPH in *E*. *coli*; all samples were tested by using 6 × His mono-antibody; (**c**) Enzyme activity assay of extracellular secretion fusion protein sfGFP-MPH and N20-MPH; N20-MPH: N-terminus 20 amino acids of sfGFP fused to the N-terminal of MPH; sfGFP-MPH: sfGFP fused to the N-terminal of MPH; (**d**) SDS-PAGE annalysis of the sub-fraction of sfGFP-MPH in *E*. *coli*; (**e**) SDS-PAGE annalysis of the sub-fraction of mCherry-MPH in *E*. *coli*; (**f**) SDS-PAGE annalysis of the expression of sfGFP and scGFP in *E*. *coli*; (**g**) SDS-PAGE annalysis of the expression of sfGFP and its mutants in *E*. *coli*; sfGFP(−18) ~ scGFP: sfGFP mutants containing net charges; T: total cell protein; SP: soluable protein of the target peotein; S: supernatant of culture media; S1~S5: supernatant of culture medium after expression process and stayed at room temperature for 0~4 days; C: cytoplasmic protein; P: periplasmic protein; OM: outer membrane protein.

The lack of any secretion signal sequences at the N-terminus of sfGFP leads to a speculation that the characteristic beta -barrel structure of sfGFP might determine its extracellular secretion. One interesting finding is that sfGFP mutants lacking the N-terminal 10 (N10) or 20 (N20) amino acids both had reduced secretion ability (Fig. 3a). More than 90% of the wild-type sfGFP were secreted out of cells after 4 days cultivation, while less than 25% and 5% were secreted under the same condition for N10 and N20 mutants, respectively. From these results, we proposed that the potential leading peptide might exist in the N-terminus that mediates the extracellular secretion of sfGFP in *E*. *coli*.

In addition, anchoring the N-terminal 20 amino acids of sfGFP to the N-terminus of Methyl phosphorous hydrolase (MPH) did not lead to its secretion (Fig. 3b), and the results of enzyme activity assay also demonstrated that comparing to N20-MPH exhibiting a relatively activity of 0.005 U/mL, sfGFP-MPH presented a relatively activity of 0.81 U/mL (Fig. 3c). In addition, fusing the whole sfGFP at the N-terminus of MPH could guide the secretion of MPH to the cell media (Fig. 3d). Thus, the N-terminal 20 amino acids of sfGFP are not the leading peptide for its extracellular secretion.

Considering that sfGFP constitutes a characteristic beta-barrel structure, we speculate that the secretion ability of sfGFP might be related to its structure. Deletion of the N-terminal 10 to 20 amino acids in sfGFP probably affected the beta-barrel structure of sfGFP, thus affecting its extracellular secretion. The Western blot results also demonstrated that trans-location efficiency decreased when expressed sfGFP (N20) in *E*. *coli* (Suppl. Figure 2c). To test our hypothesis that beta-barrel structure plays an important role in extracellular secretion, the red fluorescent protein mCherry that also contained a similar beta-barrel structure was investigated. Our results indicated that the over-expressed fusion protein mCherry-MPH could be secreted into the culture medium, and cell fraction results also exhibited that the fusion protein mCherry-MPH existed in cytoplasm, periplasmic space, and outer membrane (Fig. 3e). These results thus demonstrated that the extracellular secretion process was consistent with the secretory expression process of fusion protein sfGFP-MPH. Thus, beta-barrel structure was essential for the sfGFP secretory expression in *E*. *coli*.

### The effect of net negative charge on sfGFP auto-secretion

Another characteristic of the sfGFP is its net negative charges^26^. As we known, negative charge affects the solubility of heterologous proteins, and adding net negative charges to the terminus of heterologous proteins could increase their solubility^27^. Not surprising, our analysis using the super positively charged GFP (scGFP) (contained 36 net positive charges) observed no protein secretion into the culture medium (Fig. 2a), and the scGFP also exhibited lower fluorescence intensity and solubility compared to sfGFP (Fig. 3f).These results thus suggested that the net negative charge might affect protein solubility, and supply the driving force for the extracellular secretion.

To further investigated the effect of net negative charges on protein secretion, we constructed a series of sfGFP mutants with different net charges (such as: sfGFP_(−18)_, sfGFP_(−15)_, sfGFP_(−12)_, sfGFP_(−5)_, sfGFP_(0)_, sfGFP_(2)_, sfGFP_(7)_, sfGFP_(12)_, sfGFP_(16)_, and sfGFP_(19)_). We then over-expressed these sfGFPs in *E*. *coli* strain *Rosetta Blue*. Net negative sfGFP mutants such as sfGFP_(−15)_, sfGFP_(−12)_, sfGFP_(−7)_ and sfGFP_(−5)_ obtained high trans-location property in outer membrane. From these results, net negative sfGFP mutants presented almost the same trans-location property. Interestingly, the sfGFP mutant that contains the most net positive charges presented the lowest solubility and trans-location property in outer membrane, among which sfGFP_(7)_ and sfGFP_(12)_ exhibited high solubility but low trans-location property in the outer membrane (Fig. 3g). We can obviously distinguish the different trans-location properties between the net negative and net positive sfGFP mutants. Thus, net negative charges can supply the driving force for the secretory expression of sfGFP.

### Development of a new ***E***. *coli* sf***G***FP mediated ***P***rotein ***S***ecretion ***S***ystem (EGPSS)

The auto-secretion of sfGFP in *E*. *coli* was an interesting property that it might be used for the heterologous protein secretory expression in *E*. *coli*. To test this hypothesis, a new *E*. *coli* sf*G*FP mediated *P*rotein *S*ecretion *S*ystem (EGPSS) was developed, in which various proteins ranging from polypeptide to protein complex were fused to the C-terminal of sfGFP for secretory expression purpose (Fig. 1b, and Suppl. Figure 1).

**Figure 4:**
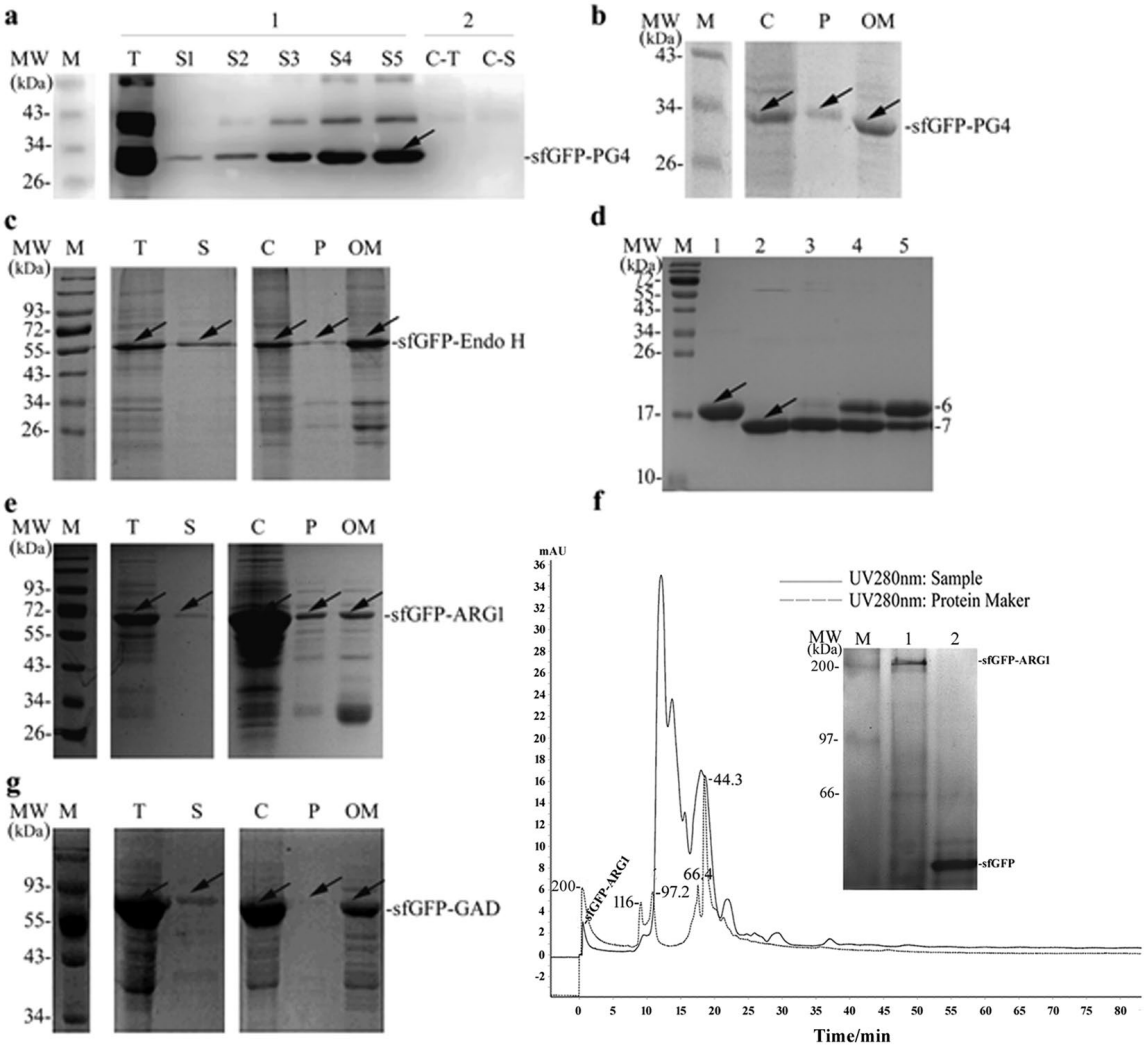
Analysis the auto secretion of sfGFP fusionsin *E*. *coli*. (**a**) Western Blot analysis of the extracellular expression of the sfGFP-PG4 in *E*. *coli*; C-T: total cell protein of the control; C-S: extracellular protein of the control; S1~S5: after expression, supernatant protein sample of culture media stayed in room temperature for one to five days; (**b**) SDS-PAGE analysis of the sub-fraction of the sfGFP-PG4 in *E*. *coli*; (**c**) SDS-PAGE analysis of the sub-fraction of sfGFP-Endo H in *E*. *coli*; (**d**) Enzyme activity analysis of sfGFP-Endo H; 1: native protein Rnase B; 2: native protein Rnase B digested by the recombinant protein Endo H (New England Biolab); 3~5: native protein Rnase B digested by sfGFP-Endo H (dilution 1:100, 1:1000, 1:10000); (**e**) SDS-PAGE analysis of the sub-fraction of sfGFP-ARG1 in *E*. *coli*; (**f**) Exclusion chromatography and SDS-PAGE analysis of sfGFP-ARG1; Lane 1: un-denatured sample of fusion protein sfGFP-ARG1; Lane 2: denatured sample of sfGFP; (**g**) SDS-PAGE analysis of the sub-fraction of sfGFP-GAD in *E*. *coli*; T: total cell proteins; S: supernatant of culture media; C: cytoplasmic protein; P: periplasmic protein; OM: outer membrane protein.

Antibacterial peptide was firstly tested in EGPSS due to its small molecular weight, toxicity to the bacteria and low structural complexity. In our studies, antibacterial peptide PG4 was fused to the C-terminus of the sfGFP and expressed in *E*. *coli* strain *Rosetta Blue*. The results demonstrated that the sfGFP-PG4 fusion protein was secreted extracellularly (about 30%) with a final fusion protein concentration of 0.121 mg/mL in the culture media (Fig. 4a; Table 1). Further analysis of the cellular localization of the sfGFP-PG4 fusion protein indicated that it existed in the cytoplasm, periplasm, and outer membrane (Fig. 4b), which was consistent with the secretory expression process of sfGFP.

**Table 1:** Polypeptide and protein used in this research.

Name	MW(target protein) (kDa)	Functional structure	Secrete production (mg/mL)	Activity (U/mg)
sfGFP-PG4	2.10	N/A	0.121	—
sfGFP-Endo H	28.99	Monometric	0.471	180
sfGFP-ARG1	36.41	Trimetric	0.397	280.36
sfGFP-GAD	50.98	Hexametric	0.198	100

After sfGFP-PG4 was successfully secreted in EGPSS, the secretory ability of sfGFP was further tested by fusing with endo-beta-N-acethylglucosamindase H (EndoH, EC3.2.1.96), a monomeric protein with molecular weight of 29 kDa. Using sfGFP as a N-terminal leading protein, sfGFP-Endo H fusion protein was successfully expressed and secreted out in *Rosetta Blue* cells (about 60%), with a protein concentration of 0.471 mg/mL in the culture media (Fig. 4c; Table 1). In addition, the sfGFP-Endo H fusion protein was also detected in the cytoplasm, periplasmic space, and outer membrane, which suggest the similar secretory mechanism of sfGFP-Endo H assfGFP-PG4 and sfGFP (Fig. 4c). It was worthy noting that sfGFP-Endo H fusion protein maintained relatively enzyme activity of 180 U/mg (Fig. 4d). Based on these results, it can be concluded that sfGFP could mediate the secretory expression of larger molecular weight protein in *E*. *coli*.

Next, more complex protein, such as human arginase-1 (ARG1), was tested in the EGPSS to evaluate the secretory property of sfGFP. Human ARG1 is a key enzyme in the urea cycle, which is regarded as a potential therapeutics against cancer. Unfortunately, heterologous expression of human ARG1 in *E*. *coli* will form inclusion bodies and is difficult to be purified^28^. In this study, the sfGFP-ARG1 fusion protein was successfully expressed and about 10% protein secreted in a soluble form through EGPSS (Fig. 4e). The concentration of the secreted sfGFP-ARG1 fusion protein in the culture media was around 0.397 mg/mL (Table 1), and the fusion protein was detected in cytoplasm, periplasmic space, and outer membrane (Fig. 4e).

One thing needs to be pointed out is that human ARG1 is a multiform protein that only exhibits enzymatic activity in a homo-trimetric form. The enzymatic activity of the secreted sfGFP-ARG1 fusion protein against L-Arg substrate was determined to be 280.36 U/mg. The homo-trimetric complex was confirmed by FPLC analysis, human Arginase 1 presented a homo-trimetric structure with the molecular weight of mono fusion protein of 63.1 kDa, and this indicated a predicted homo-trimetric fusion protein of 189.3 kDa. As it shown in the inserted Native-PAGE in Lane 1, the molecular weight of the fusion protein was consisted with the predicted one, indicating a homo-trimetric structure of sfGFP-ARG1 during the secretory process (Fig. 4f). In addition, the detection of sfGFP-ARG1 fusion protein in the outer membrane suggested that the recombinant strain could act as ‘bioreactor’ with the surface immobilized sfGFP-ARG1. Actually, the whole cell enzyme activity results confirmed our hypothesis that the sfGFP-ARG1 fusion protein immobilized on the outer membrane exhibited an enzyme activity of 6.91 ± 0.019 U/OD_600nm_ against L-Arg.

Although the successful expression and secretion of PG4, Endo-H, and human ARG1 showed the advantage of the EGPSS, it is still unknown whether complex proteins can be applied with the EGPSS. Glutamate decarboxylase (GAD, EC4.1.1. 15) catalyzes the synthesis of gamma-aminobutyric acid (GABA) from glutamic acid or glutamate. It is a 50.98 kDa protein that has to form a homo-hexametric complex with co-factor pyridoxine phosphate (PLP) to perform its catalytic activity^29^. We fused GAD to the C-terminus of sfGFP for its secretion expression in *Rosetta Blue* cells. The results clearly indicated that the sfGFP-GAD fusion protein could be secreted into the culture medium (about 10%) with a protein concentration of 0.198 mg/mL. The sfGFP-GAD fusion protein was also detected in the cytoplasm, periplasmic space, and outer membrane, similar to other proteins tested in this study (Fig. 4g). Moreover, the glutamate decarboxylate activity was detected in the culture media as 100 U/mg (Table 1), which indicated that the secreted sfGFP-GAD fusion protein formed a homo-hexametric structure.

**Figure 5:**
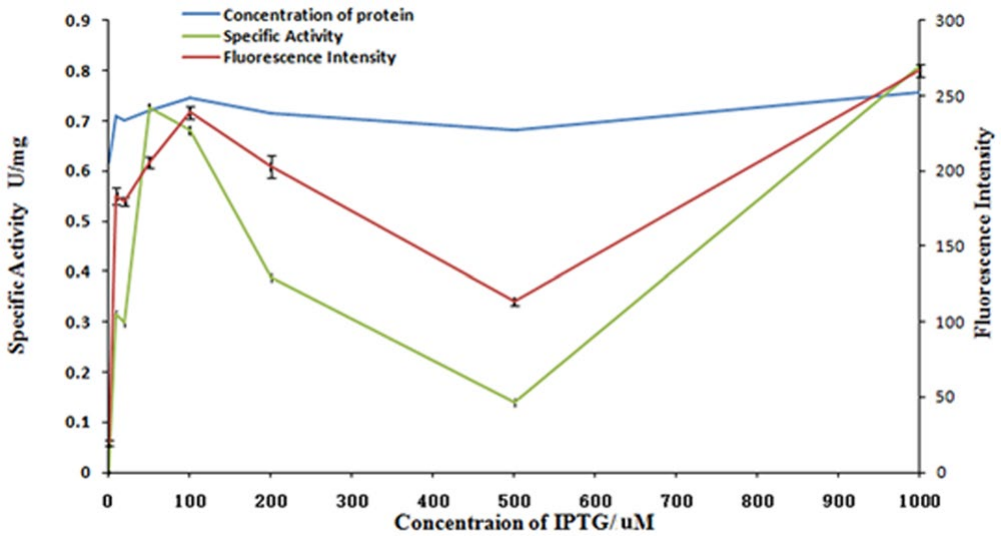
Optimazation of the extracellular secretion of sfGFP-MPH in *E*. *coli*. Blueline stands for theconcetration of extracellular secretion sfGFP-MPH;Green linestands for the special activity of extracellular secretion sfGFP-MPH; and Red line stands for the fluorescence intensity of extracellular secretion sfGFP-MPH.

Another advantage for using sfGFP fusion proteins is that it provides an easy way to quantitate the secretion levels of fusion proteins through detecting the fluorescence intensity in the cell culture media simply. According to this principle, the expression condition of heterologous protein could be easily optimized based on the secreted protein, which was quantitated by evaluating the GFP fluorescence intensity in the cell culture media. As an example, sfGFP-MPH fusion protein was optimized using this strategy in *E*. *coli*, and the best protein secretion condition of sfGFP-MPH was at 37 °C, with an IPTG final induction concentration of 100 μM (Fig. 5).

### Fed-batch production of the sfGFP-ARG1 fusion protein

**Figure 6:**
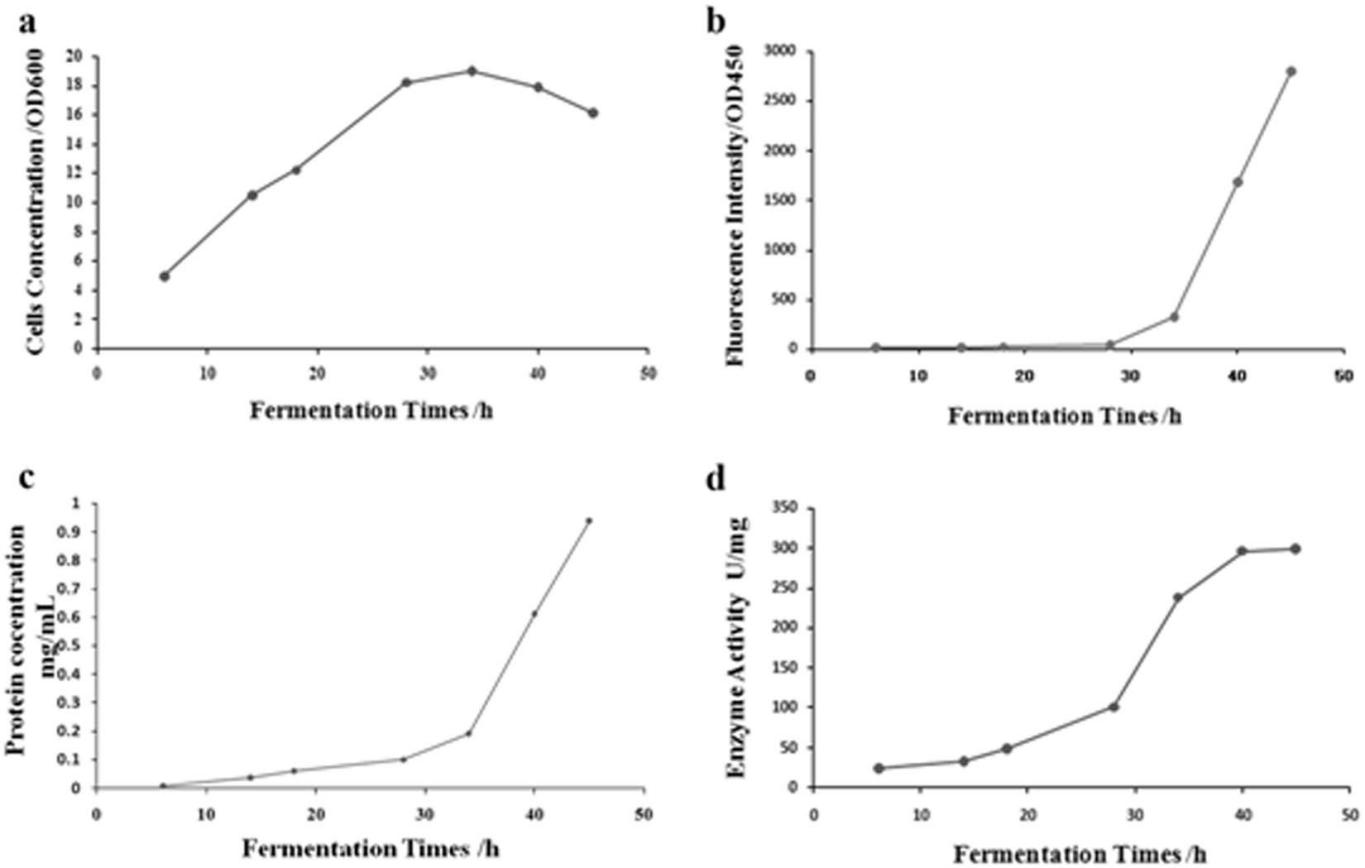
Analysis the extracellular secretion of sfGFP-ARG1 in fed-batch fermentation condition. (**a**) Cells concentration in fed-batch fermentation condition; (**b**) Fluorescence intensity of extracellar secretion sfGFP-ARG1 in fed-batch fermentation condition; (**c**) Protein concentration of extracellar secretion sfGFP-ARG1 in fed-batch fermentation condition; (**d**) Enzyme activity of extracellar secretion sfGFP-ARG1 in fed-batch fermentation condition.

Protein secretion without cell lysis is advantageous for industrial heterologous protein production, which will simplify the purification process as well as lower the production cost. In this study, EGPSS has been developed and proven to be an efficient approach for heterologous protein expression and secretion in *E*. *coli* in the shake flask. To evaluate whether EGPSS is a promising strategy for industry production, the sfGFP fusion protein secretion in the fed-batch condition was further investigated, and the secreted sfGFP-ARG1 fusion protein in the culture media was detected during the whole fed-batch fermentation process (Fig. 6).

As the fermentation continued, the cell density increased to reach its maximum after 34 hours post-inoculation, and then, began to decrease (Fig. 6a). Thus, after 34 hours fed-batch fermentation, cell lysis started. We proposed that the auto-secretion process was ended in 34 hours. Comparably, the sfGFP fluorescence was quickly increased after 28 hours post-inoculation, and kept increasing during the whole fermentation process (Fig. 6b). The fluorescence intensity of the sfGFP in the culture media was 12.2 (OD_450nm_) at the initial stage of fermentation, which increased about 26 folds to be 320.58 (OD_450nm_) at the final stage of fermentation (Fig. 6b; Table 2). In accordance with the enhanced GFP fluorescence intensity, the extracellular expression level of sfGFP-ARG1 as well as its arginase activity increased up to 0.19 mg/mL and 237.92 U/mL, respectively (Fig. 6c,d). The original concentration of fusion protein sfGFP-ARG1 was 0.006 mg/mL in the culture medium after fermentation for 6 hours, and finally with the concentration of 0.19 mg/mL after 34 hours’ fermentation, which exhibited an increased expression level of 31 folds approximately (Fig. 6c; Table 2; Suppl. Figure 3). Along with the dramatically increased protein levels, the enzyme activity of the fusion protein sfGFP-ARG1 in the culture media enhanced 10 fold from 23.78 U/mg to 237.92 U/mg during the fermentation process (Fig. 6d; Table 2). It has to be emphasized that the cell density only increased around 4-fold in the fermentation process, with the OD_600nm_ values at the initial and final stages of fermentation being 4.95 and 19, respectively (Fig. 6a; Table 2). The relative constant cell density indicated that the increased sfGFP-ARG1 levels in the cell culture media was mainly caused by the enhanced extracellular secretion of sfGFP-ARG1.

**Table 2:** sfGFP-ARG1 fusion protein in fed-batch fermentation.

Time/(h)	6	14	18	28	34	40	45
Cell concentration (OD_600nm_)	4.95	10.52	12.22	18.2	19	17.9	16.15
Fluorescence intensity (OD_450nm_)	12.21	16.95	21.01	41.49	320.58	1681.14	2805.72
Protein concentration (mg/mL)	0.006	0.036	0.06	0.09	0.19	0.61	0.94
Enzyme activity (U/mL)	23.78	32.64	48.2	100.6	237.92	296.22	299.25

## Discussion

Since the identification of GFP in 1960s, it has been engineered for applications in academia, industry, and medicine^30^. sfGFP was one GFP mutant that folds fast and well even when fused with poorly folded polypeptides. Compared to other fusion tags such as MBP and GST, sfGFP has a smaller molecular weight, but still provides good solubility to largely facilitate the folding of the fusion partner^24^.

In this study, the strong extracellular secretory property of sfGFP was identified by its ability of facilitating the secretion of various proteins fused at its C-terminus, including antibacterial peptide PG4, Endo H, human ARG1, and GAD (Fig. 1b). More interestingly, the secreted sfGFP-ARG1 and sfGFP-GAD fusion proteins can form the active trimetric and hexametric complex respectively, which indicates that the fusion of sfGFP does not affect the functional structure of ARG1 and GAD (Fig. 4; Table 1). After analyzing the different sub-cellular fractions, it suggested that the sfGFP guided secretory process could be divided into three steps: the inner membrane trans-location, the outer membrane anchoring, and the outer membrane releasing (Fig. 1a).

The fluorescence assay indicated that sfGFP was well folded during its whole transportation process, including in cytoplasm, periplasmic space, and outer membrane (Fig. 2f), which differentiated itself from the Sec pathway. Although sfGFP possesses the beta-barrel structure similar to the type Va auto-transporter proteins, the lack of N-terminal signal peptide suggested its unique secretory mechanism. One possibility was that sfGFP preceded its secretion using Tat pathway, as it has the capability of transporting folded proteins across the inner membrane. However, our further studies indicated that the cytoplasmic membrane trans-location process of sfGFP was not affected in the *tatABC* knockout *E*. *coli* strain (Suppl. Figure 2d), and the secretory mechanism of sfGFP was also different from the Tat pathway.

Unlike the N-terminus of Cel-CD, which plays a crucial factor and could mediate target heterologous protein secretion by itself^18^, the N-terminal region of sfGFP maintains its beta-barrel structure, since the truncated sfGFP with this region deletion exhibited significant low green fluorescence (data not shown), and inability to mediate heterologous protein MPH secretion by itself (Fig. 3b,c). Furthermore, the red fluorescent protein mCherry with similar beta-barrel structure can also guide heterologous protein secretion extracellularly (Fig. 3e). Combining all these results, we proposed that sfGFP carried out its extracellular secretion through a unique mechanism, in which the sfGFP’s characteristic beta-barrel structure guided its extracellular secretion. Besides the beta-barrel structure, the net negative charges of sfGFP also plays critical role in its extracellular secretion. Comparing to sfGFP that contains 7 net negative charges, the secretory ability of scGFP that contained 36 net positive charges was abolished (Fig. 2a). In fact, scGFP exhibits lower fluorescence intensity and much less solubility than those of sfGFP^26^ (Fig. 3f). Series of sfGFP mutants also proved that net negative charges of sfGFP also played a critical role in its extracellular secretion (Fig. 3g). Beta-barrel structure along with net negative charge together facilitated the sfGFP extracellular secretion in *E*. *coli*.

As we known, wtGFP also has beta-barrel structure, but over expressed wtGFP is accumulated in the cytoplasm^31^. Furthermore, adding negatively charged polypeptides (3 × Glu and 6 × Glu, named as wtGFP(3E) and wtGFP(6E), respectively) to the C-terminus of the wtGFP, almost have no effect in wtGFP’s solubility (Suppl. Figure 2e). However, releasing from outer membrane into the culture of wtGFP(3E) and wtGFP(6E) was observed while wtGFP almost have no changes from the cell fraction results (Suppl. Figure 2f). We therefore proposed that beta-barrel structure protein containing enough net negative charges help the extracellular secretion in *E*. *coli*.

It has been reported that sfGFP can be used as a fusion tag to facilitate the folding of heterologous proteins in *E*. *coli* ^24^, thus the secretory property of sfGFP expanded its application in heterologous protein expression. Based on the auto-secretion characteristic of sfGFP, a new *E*. *coli* secretion system, EGPSS, was established, in which sfGFP was fused to the N-terminus of heterologous proteins to lead the folding and secretion of the protein complex, including the sfGFP-PG4, sfGFP-Endo H, sfGFP-ARG1, and sfGFP-GAD fusion proteins.

The commercial Endo H proteins were obtained via heterologous expression in *E*. *coli* ^32^. Previous reports showed that the Endo H expression in *E*. *coli* using the P_L_ promoter of λ phage could lead to the secretion of the Endo H into the periplasmic space^32^. It was also reported that only approximately 23 mg (6000 U) of recombinant Endo H could be purified from 4 L cell culture^33, 34^. However, using the EGPSS in this study, the sfGFP-Endo H fusion protein was successfully secreted out of the cells into the culture medium, with a concentration of 0.471 mg/mL. Furthermore, fusion protein sfGFP-Endo H also exhibited the enzyme activity of 180 U/mg (Table 1).

Other than peptide and normal size protein, such as PG4 and Endo H, EGPSS was also successfully applied in the secretion of proteins with complex conformations, such as the trimetric human ARG1 and hexametric GAD. As a prospective pharmaceutical drug, arginase has been widely studied, including its heterologous expression in *E*. *coli* cells. In this research, sfGFP-ARG1, as soluble form, was successfully expressed and auto-secreted using the EGPSS (Fig. 4e). More interestingly, the outer membrane anchored and extracellular secreted human ARG1 can form functional homo-trimetric complex, the secreted sfGFP-ARG1 fusion protein can reach a concentration of 0.397 mg/mL, with a specific activity of 280.36 U/mg against L-Arg (Fig. 4e; Table 1). Compared with secreted expression by the *P*. *pastoris* and purified by Ni^2+^-affinity chromatography (248.4 U/mg)^35^, EGPSS provides higher enzyme activity, and at the same time shortens the time consuming protein purification process. Simultaneously, the sfGFP-ARG1 fusion protein immobilized on the *E*. *coli* outer membrane could turn the recombinant *E*. *coli* strain into a ‘bioreactor’, with an enzyme activity of 6.91 ± 0.019 U/OD_600nm_.

Besides human ARG1, a complex protein GAD was also successfully applied using EGPSS. The sfGFP-GAD fusion protein was expressed and secreted into the culture medium with a concentration of 0.198 mg/mL (Fig. 4g; Table 1). It was also noticed that the cofactor PLP could be covalently bound to the GAD, facilitating the formation of the homo-hexametric structure, which presented a specific activity of 100 U/mg against L-Glu (Table 1).

To evaluate the possibility whether EGPSS is a promising strategy for industrial applications, the efficiency and stability of EGPSS was also investigated in the fed-batch condition by expressing the sfGFP-ARG1 fusion protein in a 5-L fermenter. The concentration of the extracellularly secreted sfGFP-ARG1 fusion protein researched 0.19 mg/mL, with a specific activity of 237.92 U/mg against L-Arg (Table 2). The increased level of the secreted sfGFP-ARG1 indicates the efficiency and stability of EGPSS in fed-batch condition, potentially providing an easy method for commercial heterologous protein production.

In summary, sfGFP secretory expression in the *E*. *coli* strain *Rosetta Blue* was identified, which went through the inner membrane trans-location, outer membrane anchoring, and final releasing steps. We proposed that the secretion guided by sfGFP might be determined by its characteristic beta-barrel structure and net negative charges. Using the secretory property of sfGFP, an EGPSS system was developed in *E*. *coli* that different proteins, ranging from small polypeptide to protein complex could be auto-secreted into culture media through fusion to the C-terminal end of sfGFP. In addition, sfGFP fusion protein maintained the enzymatic activity of target proteins. Moreover, the immobilization of sfGFP-target protein on the cell surface could convert the recombinant cell as a ‘bioreactor’ for whole cell catalysis. This study thus establishes a new strategy in the development and utilization of sfGFP.

## Methods

### Bacterial strains and plasmids

*E*. *coli* strains, *XL*-*GOLD* and *Rosetta Blue* (Invitrogen, NY, USA), were used for genetic manipulation and recombinant production, respectively. The genes of sfGFP and related heterologous proteins were driven by the T7 promoter in the plasmids pET23a-T (designed by our research group with altered multiple cloning sites) or pET30a (Novagen, Darmstadt, Germany) (Fig. 1b; Suppl. Figure 1).

### Culture conditions

The recombinant *E*. *coli* strain *Rosetta Blue* cells harboring each recombinant plasmid were cultured in LB medium containing 50 g/mL kanamycin at 37 °C for 12 hours. When the OD_600nm_ of cell culture reached 0.5, expression of recombinant protein was induced by adding isopropy-β-D-thiogalactopyranoside (IPTG) to a final concentration of 100 μM.

A fed-batch cultivation was carried out at 37 °C, 600 rpm and 1 vvm of air supply in a 5 L-scale bioreactor (Qi Rui, Shanghai, China) containing 1-L SOB medium with 10 mM MgCl_2_. Feed used in fed-batch for continuous feeding consisted of 200 g/L yeast extract, 67 g/L glycerol, 10 g/L lactose, 10 mM, and 50 μg/mL kanamycin. The fermentation process was divided into two periods, culturing period and inducing period. The culturing period was lasted for about 18 hours in the fermentation, and then the feed was added into the fermentation culture with rate of 2 mL/min.

### Cellular fraction

After cell culturing, the culture broth was centrifuged at 5,000 rpm for 10 min for cell collection. The cell pellets were washed with PBS buffer (pH 8.0) for three times, followed by resuspended in PBS buffer (pH 8.0). The cellular proteins were extracted according to the rapid isolation method^36^. All samples were analyzed using SDS-PAGE, or western blot against the 6 × His epitope tag or the specific endogenous proteins. Samples used for the evaluation in the supernatant were unpurified and not concentrated and 20 μL of each samples were loaded. In Supplementary Figure 3b, the samples were purified proteins, and 5 μL of each samples were loaded.

### Protein purification and Enzymes assay

Recombinant sfGFP fusions presented in the culture medium were collected and purified with the 6 × His epitope tag mediated purification procedure. The supernatant of culture broth was mixed with Ni-NTA resin, followed by buffer wash, 6 × His epitope tag binding buffer with a liner gradient of imidazole from 30 to 500 mM was then continuously flowed into the column. The proteins eluted from the column were collected and analyzed. To detect the amount of proteins in supernatant fraction, we used the purified fusion proteins.

The hydrolytic activity of Endo H was assayed based on a published method using RNase B as the substrate^37^. The activity of Human ARG1 was assayed based on the Chinard reaction method using L-Arg as the substrate^38^. The activity of glutamate decarboxylase was determined according to the previously published Berthetot reaction method using L-Glu as the substrate^39^. The activity of N20-MPH and sfGFP were measured by using OP pesticide as substrates. Hydrolysis of paraoxon was measured spectrophotometrically by monitoring the production of p-nitrophenol at 405 nm with spectrophometer. The standard enzyme activity assay was carried out using 100 mM phosphate buffer (pH 7.4) supplemented with 0.2 mM substrate and 100 μL of supernatant fraction at 30 °C. Activities were expressed in units (1 μmol substrate hydrolyzed per min) per mL^40^.

## Electronic supplementary material

Supplementary Information
