# Supplementary material for: Non-peptide guided auto-secretion of recombinant proteins by super-folder green fluorescent protein in Escherichia coli
Source: Sci Rep. 2017 Aug 1;7:6990. doi: 10.1038/s41598-017-07421-3 (PMC5539203; doi:10.1038/s41598-017-07421-3)
Supplement: Supplementary file 1 — Supplementary Information [file 41598_2017_7421_MOESM1_ESM.pdf]

Non-peptide guided auto-secretion of recombinant proteins by super-folder green fluorescent protein in *Escherichia coli*

Zhen Zhang[1], Rongxing Tang[1], Dewu Zhu[1], Wenfeng Wang[1], Li Yi[1], Lixin Ma[1*]

[1]Hubei Collaborative Innovation Center for Green Transformation of Bio-resources, Hubei Key Laboratory of Industrial Biotechnology, College of Life Sciences, Hubei University, Wuhan, People's Republic of China

*Corresponding author

Prof. Lixin Ma

Department of Biochemistry and Molecular Biology

E-mail addresses: malixing@hubu.edu.cn

Supplementary Figures

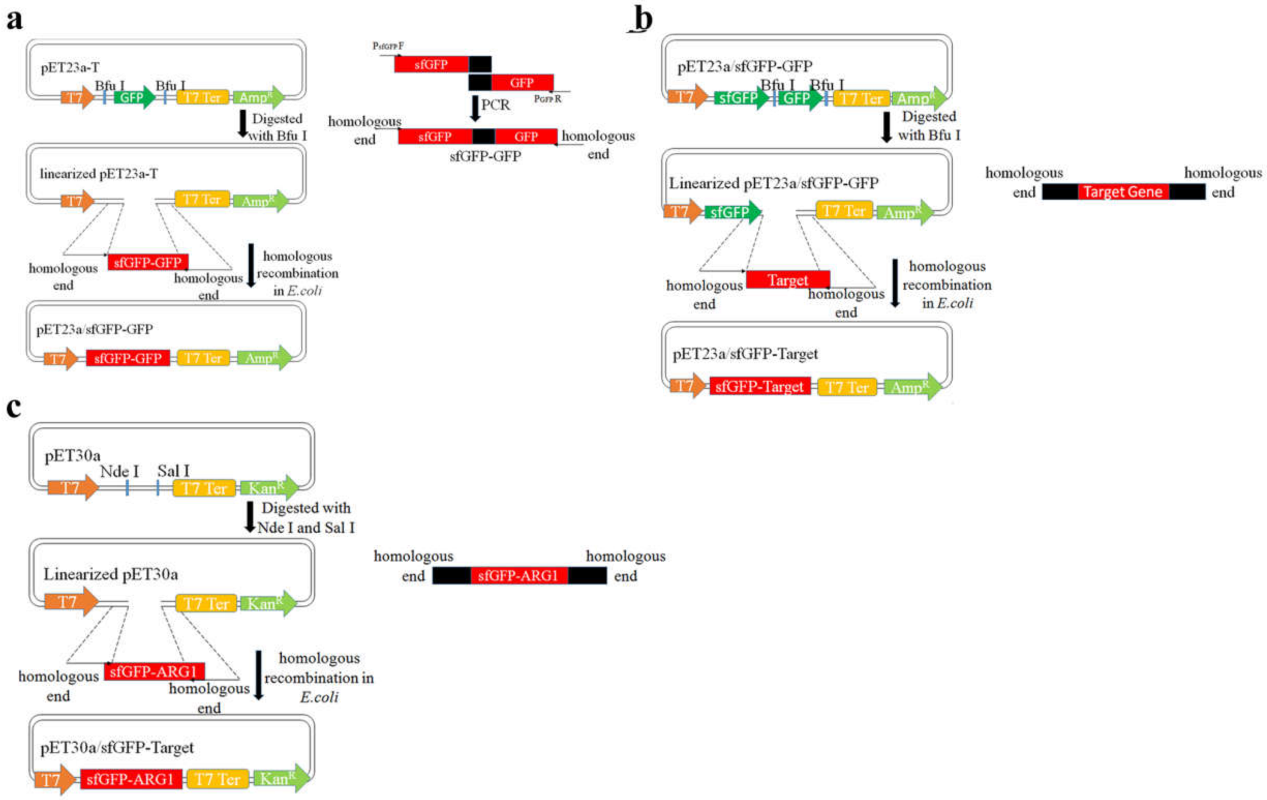

Supplementary Figure 1. Constructs in the study. (a) Schematic diagram of pET23a/sfGFP-GFP vectors; (b) Schematic diagram of pET23a/sfGFP-Target vectors; (c) Schematic diagram of pET30a/sfGFP-ARG1 vectors.

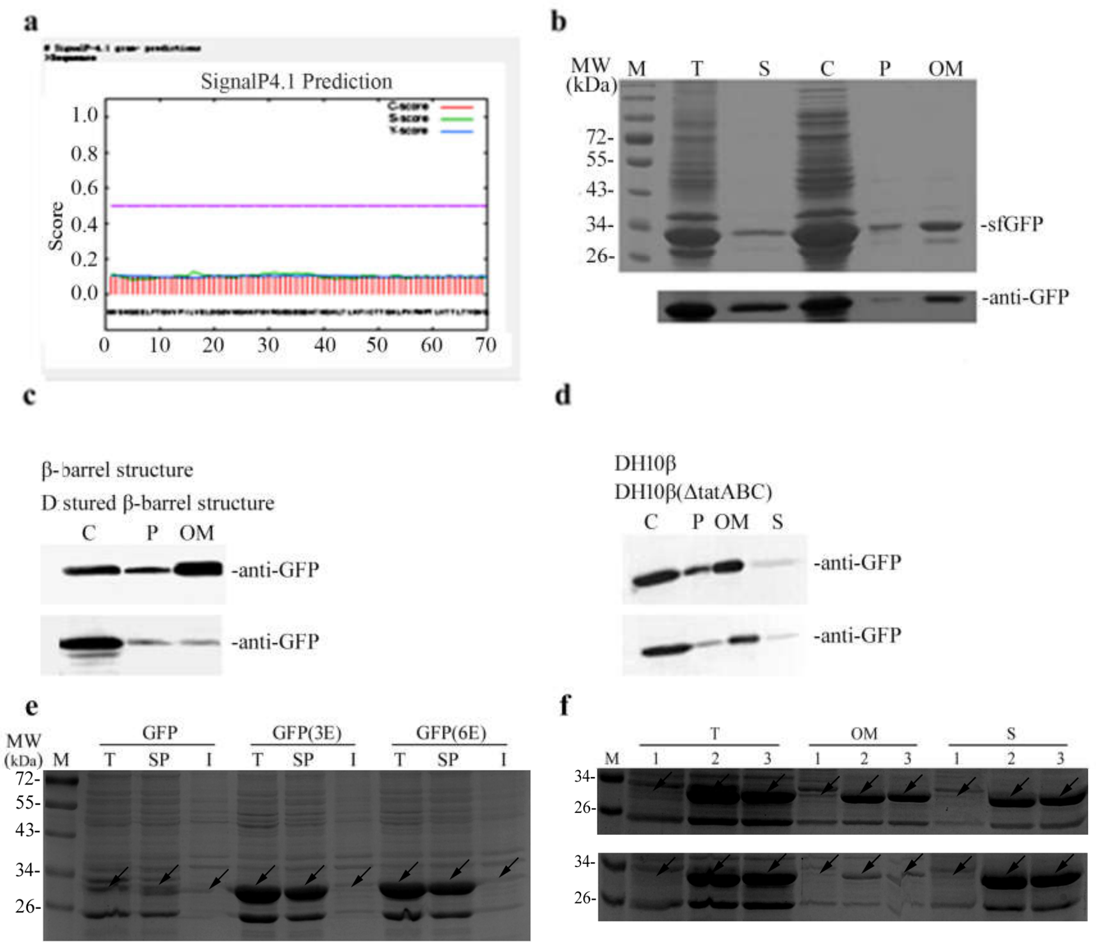

Supplementay Figiure 2. sfGFP analysis in this study. (a) Signal peptide prediction of sfGFP; (b) SDS-PAGE and Western blot analysis of the expression of sfGFP (without His-Tag) in *E. coli*(*Rosetta Blue*); (c)Western blot annalysisof the secretion of N-terminal sequence deleted sfGFP in *E. coli*(*Rosetta Blue*); (d) Western blot annalysis of the auto-secretion expression of sfGFP in DH10β and DH10β(Δ*tatABC*) strain; (e) SDS-PAGE annalysis of the expression of wtGFP and wtGFP mutants in *E.coli*(*Rosetta Blue*); (f) Sub-fraction analysis of wtGFP and wtGFP mutants in *E.coli*(*Rosetta Blue*). lane 1: wtGFP; lane 2: wtGFP(3E); lane 3: wtGFP(6E); T: total cell protein; SP: soluable part of total cell protein; I: insoluable part of total cell protein; S: extracellualr proten; C: cytoplasmic protein; P: periplasmic protein; OM: outer membrane protein.

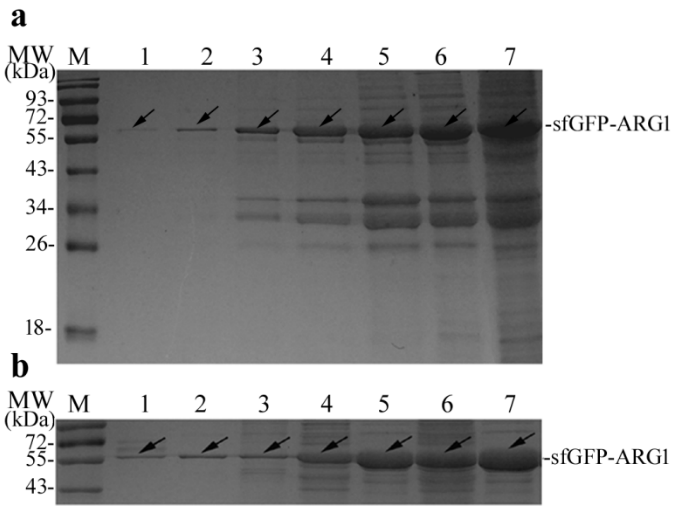

Supplementay Figiure 3. SDS-PAGE analysis extracellular secretion of sfGFP-ARG1 in fed-batch fermentation condition. (a) SDS-PAGE analysis of unpurified sfGFP-ARG1; lane 1: unpurified supernatant sample after 6 hours fermentation; lane 2: unpurified supernatant sample after 14 hours fermentation; lane 3: unpurified supernatant sample after 18 hours fermentation; lane 4: unpurified supernatant sample after 28 hours fermentation; lane 5: unpurified supernatant sample after 34 hours fermentation; lane 6: unpurified supernatant sample after 40 hours fermentation; lane 7: unpurified supernatant sample after 45 hours fermentation;each sample was loaded with 20 μL; (b). SDS-PAGE analysis of purified fusion sfGFP-ARG1; lane 1: purified supernatant sample after 6 hours fermentation; lane 2: purified supernatant sample after 14 hours fermentation; lane 3: purified supernatant sample after 18 hours fermentation; lane 4: purified supernatant sample after 28 hours fermentation; lane 5: purified supernatant sample after 34 hours fermentation; lane 6: purified supernatant sample after 40 hours fermentation; lane 7: purified supernatant sample after 45 hours fermentation; each sample was loaded with 5 μL.
